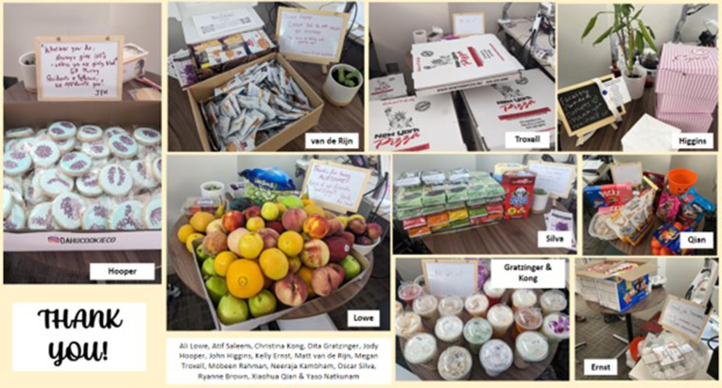# Making housestaff feel at home: impact of workspace interventions on anatomic pathology trainee wellness

**DOI:** 10.1016/j.acpath.2025.100170

**Published:** 2025-04-08

**Authors:** Kelly Ernst, Christine Heisen, Minami A. Tokuyama, Krishna L. Bharani

**Affiliations:** Department of Pathology, Stanford University, Palo Alto, CA, USA

**Keywords:** Belonging, Growth mindset, Medical education, Well-being, Workroom environment

## Abstract

To improve trainee well-being in a healthcare setting, we implemented changes shown to significantly improve employee well-being in corporate settings in an anatomic pathology (AP) trainee workspace at one large academic program and measured changes in trainee stress and well-being. An IRB-approved survey was distributed to trainees before and after implementation of workspace modifications, which included deep cleaning of the physical space, making storage space for personal items, improving access to perishable and nonperishable foods, arranging equipment to facilitate ergonomic use, and providing real and artificial visuals of nature. The survey incorporated evidence-based scales including the Ambient Belonging Scale (ABS), the five-item WHO-5 Well-Being Index, the five-item modified Spielberger State and Trait Anxiety Scales, and the Growth Mindset Scale. Pre-intervention (n = 21) and post-intervention (n = 18) participants had scores consistent with a growth mindset, no significant anxiety state or trait, and above average sense of well-being. Compared with pre-intervention survey results, post-intervention AP residents who actively worked in the space had a significantly increased sense of belonging. Free-text feedback indicated that our efforts to improve the environment and to increase access to food positively impacted their well-being as AP trainees. We show that workspace interventions implemented at our institution significantly increased a sense of belonging for our trainees independent of their growth mindset, anxiety state or trait, and sense of well-being, which was high pre- and post-intervention. These simple and cost-effective workspace interventions can be implemented broadly to create a more supportive, inclusive environment for pathology trainees.

## Introduction

Burnout, distress, and depression are more prevalent in physician trainees compared with age-matched peers, and they peak during their training as residents and fellows.^1^, ^2^, ^3^, ^4^, ^5^ Addressing trainee well-being is crucial and changes need to be implemented at an institutional level.^6^ Workspace enrichments such as the incorporation of nature and improved desk ergonomics have been shown to significantly improve employee well-being, creativity, and cognitive performance in corporate settings,^7^, ^8^, ^9^, ^10^ and have demonstrated potential in healthcare settings.^11^^,^^12^ Beyond the individual benefits of improving employee well-being and reducing burnout, workplace enrichment is related to institutional innovation, reduced turnover, and increased organizational productivity.^13^

In anatomic pathology (AP), residents and fellows (hereto referred together as ‘trainees’) spend at least two years of their adulthood in relatively isolated desk spaces. More specifically, trainees covering AP services frequently sit in cubicle arrangements at desk spaces outfitted with the bare minimum of required equipment including a microscope and computer station. The responsibility of customizing these spaces for physical and mental comfort is left to the trainee. However, the demanding schedule and temporary ownership of their space discourages trainees from investing their time and resources to enrich their workspace environment. The extent to which environment enrichments contribute to a trainee’s ability to improve well-being, fight burnout, and operate effectively as a physician and learner is not fully understood.

Contemporary studies report burnout among pathology trainees ranges between 30 and 40% with the highest rates of burnout occurring during the first year of residency training.^1^, ^2^, ^3^ Interventions including wellness education curricula and mentorship have been reported to positively impact knowledge of wellness resources and wellness promoting activities.^14^ However, there is a dearth of literature describing interventions to improve pathologist-specific and pathology trainee-specific well-being.^1^^,^^2^^,^^15^ To the best of our knowledge, no specific study has used evidence-based, well-established scales to measure pathology trainee well-being, anxiety, growth mindset and sense of belonging in response to such an intervention. Therefore, we anticipated that an intervention to improve our AP trainee workspace would positively impact trainee well-being in a measurable way. Specifically, we targeted improving trainee well-being by a) creating a clean and ergonomic environment, b) fostering a sense of belonging and goodwill, and c) increasing exposure to nature. We measured our impact using well-established metrics of belonging, well-being, anxiety, stress, and growth mindset. In this paper, we share efforts of an initiative developed by our Pathology Department Trainee Wellness Committee that can be modeled by other training programs to assess and promote a sense of belonging among those on service.

## Materials and methods

In our cohort, AP trainees spend the majority of their time in an open concept–designed office space with short wall cubicles in the center. Each cubicle is outfitted with an L-desk, a chair, a computer station with dual monitors, a phone, a microscope, and a file organizer.

An IRB-approved survey was designed using free text and Likert-scale questions. Participants were asked for their post graduation year, the stage of their training (e.g. 2nd year of clinical pathology or CP2), when was the last time they worked in the AP workroom, and how long have they worked in the workroom overall. The survey also included five scales. The Ambient Belonging Scale (ABS)^16^ measures an individual’s sense of fit in an environment using 9 questions with a 6-point scale. The WHO-5 Well-Being Index^17^ measures the level of subjective well-being over the last two weeks using five questions with a 6-point scale. The short Spielberger Anxiety Inventory State (STAIS-5)^18^ measures current anxiety state and the short Spielberger Anxiety Inventory Trait (STAIT-5)^19^ measures internal anxiety traits by asking how much people agree with five statements on a 4-point scale each. The Growth Mindset Scale^20^^,^^21^ measures how much people believe that they can get smarter if they work at it using four questions with a six-point scale. People with a ‘growth mindset’ believe that they can improve with effort, while those with a ‘fixed mindset’ believe that their effort does not change the ability they are born with. Additional open-ended feedback was elicited regarding how the work environment impacts their well-being at the end of the survey (Supplemental Material 1). This survey was distributed to AP trainees before and after implementation of workspace modifications previously shown to improve well-being in corporate settings. Specifically, the survey was emailed to trainees in June 2023, four weeks before implementing our workspace modifications, and in August 2023, six weeks after implementing our workspace modifications. To increase participation and sincerity in their answers, a unique identifier for each survey response was not collected.

**Fig. 1:**
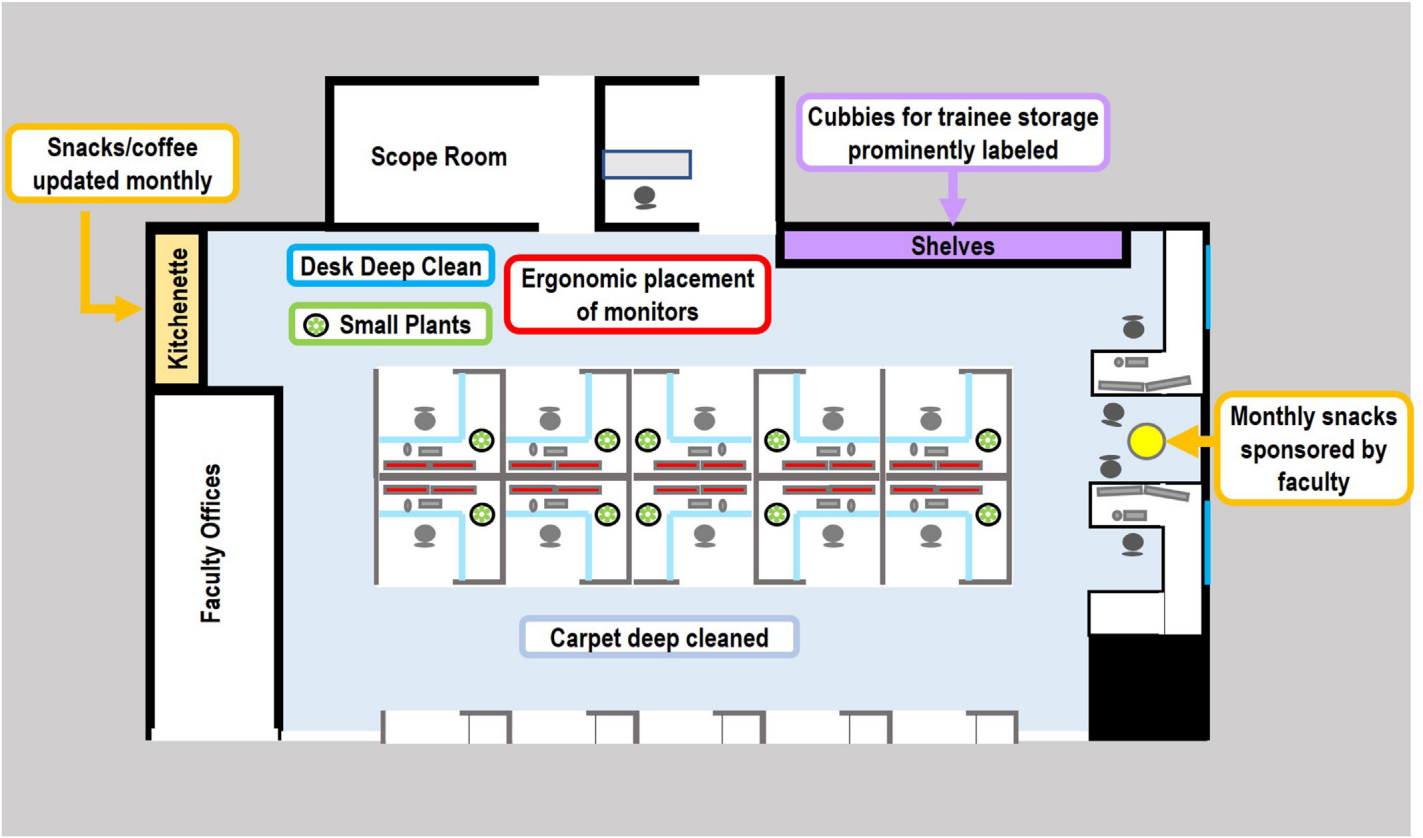
Visual summary of workspace interventions implemented to improve surgical pathology trainee well-being. A clean and ergonomic environment was created through deep cleaning and rearrangement of furniture. A sense of belonging and goodwill was fostered by creating space for trainees’ personal belongings and increasing access to snacks. Exposure to nature was achieved by placing small plants at every desk.

To create a comfortable and clean space, we decluttered open shelf space and desk drawers, cleaned and disinfected counterspace and shared equipment, shampooed the carpet, and ergonomically rearranged the monitors so they reduced neck and head movements. To foster a sense of belonging, fabric cube storage boxes with the trainees’ names shown prominently on the shelves were placed near the entrance. In addition, we stocked the kitchenette with nonperishable snacks, coffee, and tea. We also solicited funds from faculty to purchase high-quality perishable foods (e.g. custom pathology–themed cookies, farm-fresh fruits, Danish treats, etc.), which were placed with an encouraging and appreciative note from an individual faculty member to the trainees (Supplemental Fig. 1). To increase exposure to nature, we hosted a succulent plant party where trainees were able to choose and pot their own succulent plant to place at their desk. Additional larger pots with succulents were placed in the room as well, and large landscape artworks were placed on the walls of the workroom. A summary of our interventions is shown in Fig. 1.

Statistical analyses were performed using GraphPad Prism version 10.3.1 for Windows (GraphPad Software, Boston, Massachusetts USA, www.graphpad.com). Study participants were divided into three groups including the following: (1) AP residents, defined as residents currently working in the AP workspace, (2) nonactive residents (NA–AP residents), defined as pathology residents who had previously but now no longer work in the AP workspace due to rotation schedules, and (3) fellows, defined as AP fellows who are currently working the in workspace. In our program, AP/CP trainees spend two years in AP (AP1 and AP2) and then two years in clinical pathology (CP) (CP1 and CP2), and NA–AP residents have completed at least one year of AP training.

## Results

**Fig. 2:**
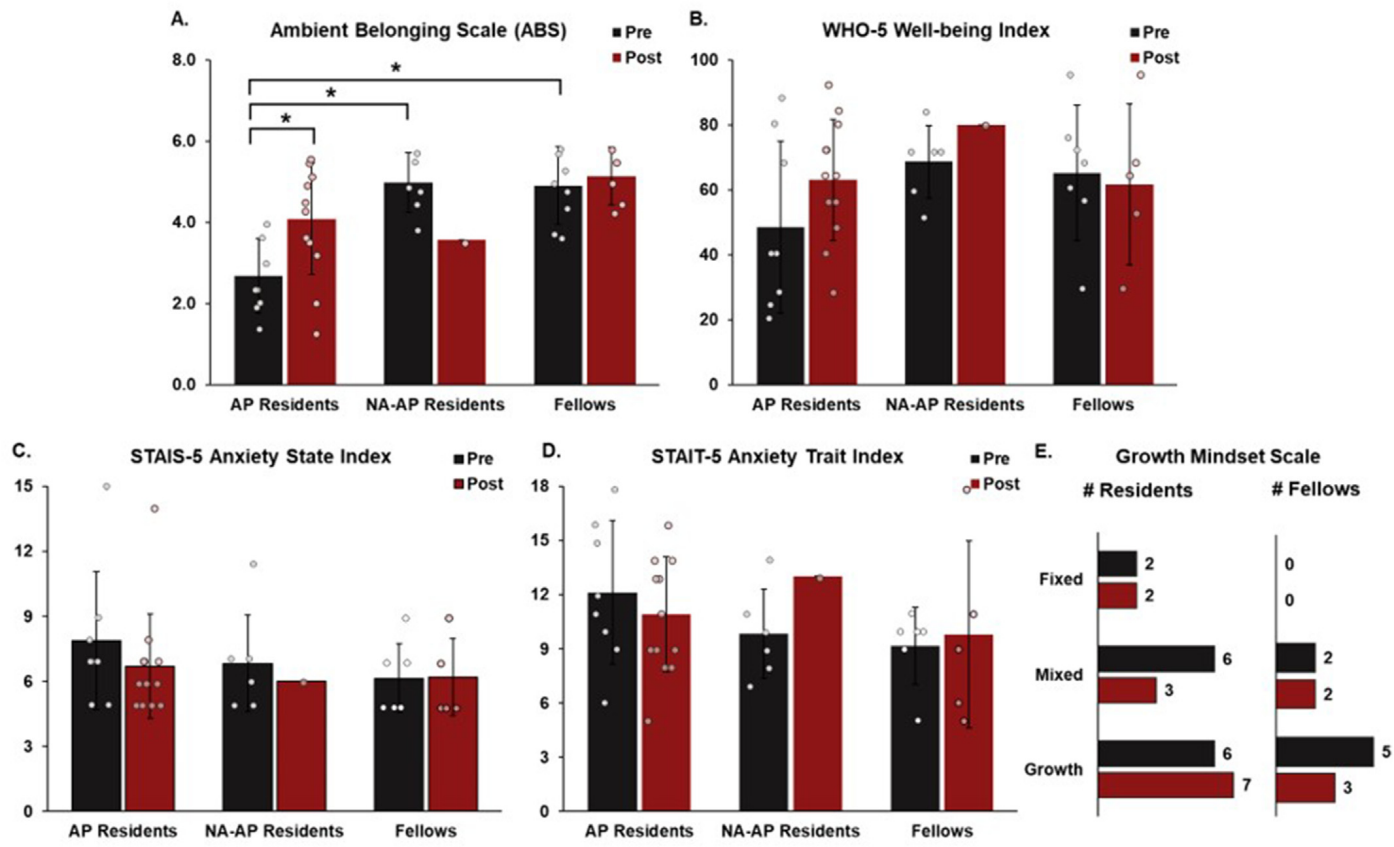
Pre- and post-intervention survey results from AP residents, NA-AP residents, and fellows. As a group, pre- and post-intervention AP residents who were actively working in the space had a lower sense of belonging (measured by ABS) than fellows who were actively working in the space (**A**). The pre-intervention AP residents also had a lower sense of belonging than the pre-intervention NA-AP residents. Compared with pre-intervention survey results, post-intervention AP residents who actively worked in the space had a significantly increased sense of belonging. There were no statistically significant differences present between groups and pre/post-intervention in measures of well-being (**B**), stress (**C** and **D**), and growth mindset (**E**). ∗*P* < 0.05. ABS: Ambient Belonging Scale; AP: anatomic pathology; NA: nonactive.

The effects of both group (AP residents, NA-AP residents, fellows) and time (pre- and post-intervention) on collected measures from the Ambient Belonging Scale (ABS), WHO-5 Well-Being Index, STAIS-5 Anxiety State Index, and STAIT-5 Anxiety Train Index were compared using two-way analysis of variance. The means and standard deviations for these measures are presented in Fig. 2A–D.

A statistically significant interaction was found between group and time on the ABS score, *F* (2, 34) = 3.56, *P* = 0.040. A significant main effect in group was present in ABS, indicating that pre- and post-intervention AP residents (n = 20, μ = 3.5, σ = 1.4) as a whole had statistically lower ABS scores than the NA-AP residents (n = 7, μ = 4.8, σ = 0.9) and fellows (n = 12, μ = 5.0, σ = 0.8), *F* (2, 34) = 9.78, *P* = 0.004. *Post hoc* testing using Tukey’s honestly significant difference (HSD) indicated that the pre-intervention AP resident group had significantly lower ABS scores than the pre-intervention NA-AP resident group (*P* = 0.0003) and the pre-intervention fellow group (*P* = 0.0003). There was no significant main effect in time, *F* (1, 34) = 0.0613, *P* = 0.806. The finding of a significant interaction and a nonsignificant main effect in time suggested our intervention did not affect all three groups equally. *Post hoc* testing using Tukey’s HSD indicated that there was a significant increase in the ABS score post-intervention in AP residents compared with pre-intervention (*P* = 0.002), which was not found in the NA-AP residents (*P* = 0.222) or Fellows (*P* = 0.715) (Fig. 2A). There was no statistically significant interaction, group main effects, or time main effects found in WHO-5 Well-Being Index, STAIS-5 Anxiety State Index, and STAIT-5 Anxiety Train Index measures (Fig. 2B–D). Fisher’s exact test was used to determine if there was a significant association between group and growth mindset pre- and post-intervention (Fig. 2E). There was no statistically significant association between the groups, pre- and post-intervention (two-tailed *P* = 0.867) in this growth mindset measure. Free-text feedback was very positive, with new access to free snacks and coffee, workspace ergonomic changes, and the addition of plants mentioned most frequently.

## Discussion

This paper describes simple workspace interventions that demonstrated a statistically significant increased sense of belonging among trainees in an anatomical pathology training workspace based on evidence-based measures of well-being. The trainees who actively worked in our space (AP residents) had a significantly lower sense of belonging compared with trainees who no longer worked in the space (NA-AP residents). Remarkably, they also had a lower sense of belonging than the fellows who recently joined the workspace. We found that this lower sense of belonging was not related to measures of well-being, stress, anxiety, or growth mindset across all three groups pre- and post-intervention. Participant scores were overall consistent with a growth mindset, no significant anxiety state or trait, and above average sense of well-being in the pre-survey (n = 21) and post-survey (n = 18). After implementing our simple workspace interventions, we were able to measure a statistically significant increase in belonging in the AP residents who indicated that our intervention positively affected their sense of belonging in anonymous free-text feedback.

Trainee sense of belonging was measured through the Ambient Belonging Score that was adapted from the Stanford ‘Social Psychological Answers to Real-World Questions’, or SPARQ. This measure was inspired by several studies that demonstrated an association between ambient environmental cues and how they signal to certain groups of individuals who belong in a space.^16^ In the earliest study of ambient belonging, the analysis showed how workroom environment impacted women’s interest in computer science.^16^ The impact of increased sense of belonging in our study among AP trainees after our workspace improvement intervention is not entirely clear. However, studies of college students highlight the power of an intervention like ours to promote improved performance, persistence, and diversity, equity, and inclusion in the literal and figurative space of pathology graduate medical education.^22^^,^^23^ Future studies to investigate the impact and significance of ambient belonging in graduate medical education is warranted.

A second interesting finding in our study is the increased sense of belonging of fellows relative to residents. Prior research shows slightly better metrics of well-being among pathology fellows relative to residents.^3^ Differences in reported sense of well-being and belonging among residents versus fellows may be due to an increased sense of utility and competency among more senior trainees. In addition, all of the fellows completed their residency training during the COVID-19 pandemic and experienced varying degrees of social distancing remote work policies. Trainees were surveyed in 2022 and 2023, after many COVID-19 pandemic restrictions were lifted and trainees were not working remotely. It is possible that the high pre-intervention scores found in fellows may reflect the transition to a more ‘normal’ workplace after the pandemic. Importantly, in our study, the sense of belonging in our trainees seemed to be independent of how novel the space was to them. All fellow study participants completed their residencies at outside institutions and recently joined our institution, so both the AP residents and fellows were equally new to the workspace.

A key component of our intervention is a sustainability plan to ensure that these changes are maintained over time. Our department has a Trainee Wellness Education Committee with a faculty lead that collaborates with the chief residents to oversee wellness education sessions and initiatives such as these. Through this committee, trainees volunteer for monthly duties such as reaching out to faculty to purchase and deliver high-quality perishable foods and coordinating with the graduate medical education administration to restock the nonperishable snack cabinet. The group meets every six to eight weeks with dedicated faculty oversight to ensure continued programming.

Our study has several limitations that should be considered to contextualize and elaborate upon our results. For one, our study results are based on a small sample size from a single institution and may not be representative. In addition, to encourage participation and sincerity of survey responses we did not collect identifying information from our participants. This precluded us from matching our pre- and post-survey findings and measuring individual changes of scores due to our interventions. Finally, our study cannot discreetly identify which one of our many interventions directly improved the trainees’ sense of belonging. Additional studies will have to be performed to deconvolute our interventions and identify the intervention having the greatest and most direct impact.

In conclusion, we encourage other academic pathology programs to consider these small but powerful changes in a workroom environment. By creating a clean and ergonomic environment, fostering a sense of belonging and goodwill through small and reliable gestures, and increasing exposure to nature, we can improve the trainee experience and try to proactively address the increased rate of burnout, distress, and depression present in physician trainees.

## Funding

This research received no specific grant from any funding agency in the public, commercial, or not-for-profit sectors.

## Declaration of competing interest

No conflict.

## Figures and Tables

**figs1:**