# Supplementary material for: Making housestaff feel at home: impact of workspace interventions on anatomic pathology trainee wellness
Source: Acad Pathol. 2025 Apr 8;12(2):100170. doi: 10.1016/j.acpath.2025.100170 (PMC12005852; doi:10.1016/j.acpath.2025.100170)
Supplement: Multimedia component 1 [file mmc1.docx]

**Supplementary Material 1**

**Pre-survey**

Thank you for reviewing the consent to our study on the effects of workroom environment on well-being.

This is the pre-survey which consists of 27 questions on a Likert scale and 5 open ended question.

Completing this survey constitutes your consent.

Please select the arrow button to go to the next page.

Page < # >

What PGY year are you?

What best describes the level of your training (please only select one)?

AP1

AP2

AP3

C1

C2

C3

Fellow

Other

When was the last time you worked in the Surgical Pathology workroom (e.g., currently working in the room, worked in the room two weeks ago, months ago, last year, 2019-2020)?

How long have you worked in the Surgical Pathology workroom during your training (e.g., two years, two months, several months last year)?

Page < # >

How much do you agree with each of the following statements in regard to the Surgical Pathology Workroom in 300p? (Please select one per statement)

|  | Strongly Disagree | Somewhat Disagree | Slightly Disagree | Slightly Agree | Somewhat Agree | Strongly Agree |
| --- | --- | --- | --- | --- | --- | --- |
| I belong in this room | o | o | o | o | o | o |
| I will be successful in this room | o | o | o | o | o | o |
| I “fit” in this environment | o | o | o | o | o | o |
| I feel welcome in the room | o | o | o | o | o | o |
| I am similar to the other people in this room | o | o | o | o | o | o |
| I enjoy spending time in this room | o | o | o | o | o | o |
| I fit in with other people in this room | o | o | o | o | o | o |
| I like the way being in this room makes me feel | o | o | o | o | o | o |

Please select the arrow button to go to the next page.

Page < # >

STAIS-5

A number of statements which people have used to describe themselves are given below. Read each statement and then circle the number at the end of the statement that indicates HOW YOU FEEL RIGHT NOW‚ that is‚ at this moment. There are no right or wrong answers. Do not spend too much time on any one statement but give the answer which seems to describe your present feelings best. Thank you.

|  | Not at all | Somewhat | Moderately so | Very much so |
| --- | --- | --- | --- | --- |
| I feel upset. | o | o | o | o |
| I feel frightened. | o | o | o | o |
| I feel nervous. | o | o | o | o |
| I am jittery. | o | o | o | o |
| I feel confused. | o | o | o | o |

Please select the arrow button to go to the next page.

Page < # >

STAIT-5

A number of statements which people have used to describe themselves are given below. Read each statement and then circle the number at the end of the statement that indicates HOW YOU GENERALLY FEEL. There are no right or wrong answers. Do not spend too much time on any one statement but give the answer which seems to describe how you generally feel. Thank you.

|  | Not at all | Somewhat | Moderately so | Very much so |
| --- | --- | --- | --- | --- |
| I feel that difficulties are piling up so that I cannot overcome them. | o | o | o | o |
| I worry too much over something that really doesn't matter. | o | o | o | o |
| Some unimportant thoughts run through my mind and bothers me. | o | o | o | o |
| I take disappointments so keenly that I can't put them out of my mind. | o | o | o | o |
| I get in a state of tension or turmoil as I think over my recent concerns and interests. | o | o | o | o |

Please select the arrow button to go to the next page.

Page < # >

WHO-Five Well-Being Index (WHO-5)

Please respond to each item by marking one box per row, regarding how you felt in the last two weeks.

|  | All of the time | Most of the time | More than half the time | Less than half the time | Some of the time | At no time |
| --- | --- | --- | --- | --- | --- | --- |
| I have felt cheerful in good spirts. | o | o | o | o | o | o |
| I have felt calm and relaxed. | o | o | o | o | o | o |
| I have felt active and vigorous. | o | o | o | o | o | o |
| I work up feeling fresh and rested. | o | o | o | o | o | o |
| My daily life has been filled with things that interest me. | o | o | o | o | o | o |

Please select the arrow button to go to the next page.

Page < # >

Read each sentence below and then circle the one number that shows how much you agree with it. There are no right or wrong answers.

|  | Strongly Disagree | Disagree | Mostly Disagree | Mostly Agree | Agree | Strongly Agree |
| --- | --- | --- | --- | --- | --- | --- |
| You have a certain amount of intelligence, and you can’t really do much to change it. | o | o | o | o | o | o |
| Your intelligence is something about you that you can’t change very much. | o | o | o | o | o | o |
| You can learn new things, but you can’t really change your basic intelligence. | o | o | o | o | o | o |

Please select the arrow button to go to the next page.

Page < # >

Please use this space to share how your current work environment in the surgical pathology resident and fellow room impacts your wellbeing as a trainee in pathology.

Please select the arrow button to go to the next page.

Page < # >

Thank you for completing this survey! As part of this study, you may receive a post-intervention survey.

If you have any questions of have felt you have been hurt by being a part of this study, please contact the Protocol Director, Dr. Kelly Ernst ([kellyernst@stanford.edu](mailto:kellyernst@stanford.edu)).

If you have any feeling of distress due to any reason, please reach out to Stanford WellConnect at [wellconnect@stanford.edu](mailto:wellconnect@stanford.edu). To contact WellConnect for day-of assistance, you can reach their staff at (650) 724-1395  — they are available 24 hours a day.

All contact with WellConnect staff is confidential. Their mission is to support the emotional well-being of Stanford physicians and faculty scientists with consultation and referrals to pre-screened, highly qualified mental health professionals.

**Post-survey**

Page < # >

Thank you for reviewing the consent to our study on the effects of workroom environment on well-being.

This is the post-survey which consists of 27 questions on a Likert scale and 7 open ended question. Completing this survey constitutes your consent.

Please select the arrow button to go to the next page.

Page < # >

Have you taken the pre-intervention survey?

What PGY year are you?

What best describes the level of your training (please only select one)?

AP1

AP2

AP3

C1

C2

C3

Fellow

Other

When was the last time you worked in the Surgical Pathology workroom (e.g., currently working in the room, worked in the room two weeks ago, months ago, last year, 2019-2020)?

How long have you worked in the Surgical Pathology workroom during your training (e.g., two years, two months, several months last year)?

Page < # >

How much do you agree with each of the following statements in regard to the Surgical Pathology Workroom in 300p? (Please select one per statement)

|  | Strongly Disagree | Somewhat Disagree | Slightly Disagree | Slightly Agree | Somewhat Agree | Strongly Agree |
| --- | --- | --- | --- | --- | --- | --- |
| I belong in this room | o | o | o | o | o | o |
| I will be successful in this room | o | o | o | o | o | o |
| I “fit” in this environment | o | o | o | o | o | o |
| I feel welcome in the room | o | o | o | o | o | o |
| I am similar to the other people in this room | o | o | o | o | o | o |
| I enjoy spending time in this room | o | o | o | o | o | o |
| I fit in with other people in this room | o | o | o | o | o | o |
| I like the way being in this room makes me feel | o | o | o | o | o | o |

Please select the arrow button to go to the next page.

Page < # >

STAIS-5

A number of statements which people have used to describe themselves are given below. Read each statement and then circle the number at the end of the statement that indicates HOW YOU FEEL RIGHT NOW‚ that is‚ at this moment. There are no right or wrong answers. Do not spend too much time on any one statement but give the answer which seems to describe your present feelings best. Thank you.

|  | Not at all | Somewhat | Moderately so | Very much so |
| --- | --- | --- | --- | --- |
| I feel upset. | o | o | o | o |
| I feel frightened. | o | o | o | o |
| I feel nervous. | o | o | o | o |
| I am jittery. | o | o | o | o |
| I feel confused. | o | o | o | o |

Please select the arrow button to go to the next page.

Page < # >

STAIT-5

A number of statements which people have used to describe themselves are given below. Read each statement and then circle the number at the end of the statement that indicates HOW YOU GENERALLY FEEL. There are no right or wrong answers. Do not spend too much time on any one statement but give the answer which seems to describe how you generally feel. Thank you.

|  | Not at all | Somewhat | Moderately so | Very much so |
| --- | --- | --- | --- | --- |
| I feel that difficulties are piling up so that I cannot overcome them. | o | o | o | o |
| I worry too much over something that really doesn't matter. | o | o | o | o |
| Some unimportant thoughts run through my mind and bothers me. | o | o | o | o |
| I take disappointments so keenly that I can't put them out of my mind. | o | o | o | o |
| I get in a state of tension or turmoil as I think over my recent concerns and interests. | o | o | o | o |

Please select the arrow button to go to the next page.

Page < # >

WHO-Five Well-Being Index (WHO-5)

Please respond to each item by marking one box per row, regarding how you felt in the last two weeks.

|  | All of the time | Most of the time | More than half the time | Less than half the time | Some of the time | At no time |
| --- | --- | --- | --- | --- | --- | --- |
| I have felt cheerful in good spirts. | o | o | o | o | o | o |
| I have felt calm and relaxed. | o | o | o | o | o | o |
| I have felt active and vigorous. | o | o | o | o | o | o |
| I work up feeling fresh and rested. | o | o | o | o | o | o |
| My daily life has been filled with things that interest me. | o | o | o | o | o | o |

Please select the arrow button to go to the next page.

Page < # >

Read each sentence below and then circle the one number that shows how much you agree with it. There are no right or wrong answers.

|  | Strongly Disagree | Disagree | Mostly Disagree | Mostly Agree | Agree | Strongly Agree |
| --- | --- | --- | --- | --- | --- | --- |
| You have a certain amount of intelligence, and you can’t really do much to change it. | o | o | o | o | o | o |
| Your intelligence is something about you that you can’t change very much. | o | o | o | o | o | o |
| You can learn new things, but you can’t really change your basic intelligence. | o | o | o | o | o | o |

Please select the arrow button to go to the next page.

Page < # >

What, if any, changes to your current work environment in the surgical pathology resident and fellow room have you recognized or appreciated?

Please use this space to share how your current work environment in the surgical pathology resident and fellow room impacts your wellbeing as a trainee in pathology.

Please select the arrow button to go to the next page.

Page < # >

Thank you for completing this survey!

If you have any questions of have felt you have been hurt by being a part of this study, please contact the Protocol Director, Dr. Kelly Ernst ([kellyernst@stanford.edu](mailto:kellyernst@stanford.edu)).

If you have any feeling of distress due to any reason, please reach out to Stanford WellConnect at [wellconnect@stanford.edu](mailto:wellconnect@stanford.edu). To contact WellConnect for day-of assistance, you can reach their staff at (650) 724-1395  — they are available 24 hours a day.

All contact with WellConnect staff is confidential. Their mission is to support the emotional well-being of Stanford physicians and faculty scientists with consultation and referrals to pre-screened, highly qualified mental health professionals.
