# Supplementary material for: Making housestaff feel at home: impact of workspace interventions on anatomic pathology trainee wellness
Source: Acad Pathol. 2025 Apr 8;12(2):100170. doi: 10.1016/j.acpath.2025.100170 (PMC12005852; doi:10.1016/j.acpath.2025.100170)
Supplement: Multimedia component 2 [file mmc2.docx]

**Supplemental Figure Legend**

**Supplemental Figure 1.** Images of provisioned food with inspirational quotations on a white board from faculty to trainees in their workspace.
